# From soluble uric acid to sodium urate crystal: immune metabolic inflammation driven by uric acid morphological transformation and mechanism-oriented therapy

**DOI:** 10.3389/fimmu.2026.1794782

**Published:** 2026-03-05

**Authors:** Qianqian Yang, Yundong Xu, Jian Zhang, Niqin Xiao, Hongting Lu, Bingbing Chen, Bo Yang, Zhaohu Xie, Zhaofu Li

**Affiliations:** 1 Yunnan University of Chinese Medicine, Kunming, Yunnan, China; 2 Zhaotong Hospital of Traditional Chinese Medicine, Zhaotong, Yunnan, China

**Keywords:** NLRP3 inflammasome, oxidative stress, programmed cell death, sodium urate crystal, soluble uric acid

## Abstract

Uric acid has complex bidirectional effects on human physiology and disease, influenced by its antioxidant capacity, metabolic regulatory roles, and pro-inflammatory properties, all of which are highly context-dependent. In this review, we synthesize recent advancements related to the continuum from soluble uric acid (SUA) to amorphous monosodium urate (AMSU) and, ultimately, to crystalline monosodium urate (MSU). We propose that AMSU may act as a transitional intermediate that connects the soluble and crystalline states. Notably, AMSU may serve as a buffering stage between crystallization and inflammatory activation, providing a conceptual bridge between urate phase transitions and immune–metabolic signaling. Building on this idea, we establish a framework that links urate state dynamics with immune-metabolic pathways and disease progression. We systematically summarize the physiological roles of SUA in maintaining redox homeostasis and regulating metabolism, and we examine how sustained hyperuricemia contributes to chronic organ damage through impaired autophagy and metabolic inflammation. Additionally, we outline how the formation of MSU crystals triggers acute inflammatory responses via the TLR–NLRP3 two-signal model. Subsequent processes, such as neutrophil extracellular traps formation and macrophage polarization, drive chronic tissue remodeling and progressive pathology. Finally, we connect these mechanistic insights to both established and emerging therapeutic strategies, emphasizing the potential value of stage-specific and mechanism-oriented interventions. By conceptualizing uric acid biology as a dynamic, multi-state process, this review offers an integrated perspective on hyperuricemia-associated diseases and suggests directions for future targeted therapeutic research.

## 1 Introduction

Uric acid (UA) possesses both antioxidant and pathogenic biological characteristics. Under normal physiological conditions, soluble uric acid (SUA) scavenges reactive oxygen species (ROS) and maintains cellular redox homeostasis (1). However, prolonged elevation of serum uric acid levels due to excessive production or limited renal excretion can cause uric acid to transition from “antioxidant molecules” to “pro-oxidants.” This shift induces oxidative stress, metabolic imbalance, and inflammatory reactions, prompting a re-evaluation of its mechanisms in multisystem diseases (2).

Research indicates that the biological effects of uric acid are closely linked to its physicochemical forms. SUA primarily exists in plasma as a monomer, regulating mitochondrial function, cell metabolism, and oxidative signal transduction. When SUA concentration exceeds the physiological saturation threshold, it forms amorphous sodium urate (AMSU), which holds biological significance. Over time, AMSU converts to monosodium urate (MSU), leading to deposition in joints, kidneys, and other tissues, resulting in inflammatory reactions (3). Studies demonstrate that the transformation of uric acid from a dissolved state to a crystalline state is not merely a passive physical deposition process, but a dynamic, multistage process regulated by local microenvironment factors such as pH, temperature, ionic strength, and extracellular matrix components (4, 5).

Therefore, clarifying the dynamic transformation between SUA and MSU, as well as the mechanisms involved in disease occurrence, is crucial for understanding the continuous pathological spectrum of uric acid-related diseases. This article systematically reviews the structural basis and biological effects of uric acid morphological changes, with a focus on the distinct mechanisms of SUA and MSU in metabolic regulation and immune inflammatory response, ultimately aiming to accurately target disease treatment based on these mechanisms.

## 2 The uric acid continuum: from soluble uric acid to monosodium urate crystals

Under physiological conditions (pH ≈ 7.4, temperature 37 °C), uric acid primarily exists in body fluids as monourate ions, referred to as SUA. This compound serves not only as an excretable waste but also as one of the main antioxidants in plasma.

When the concentration of uric acid in blood and synovial fluid exceeds physiological solubility, SUA transitions into a thermodynamically supersaturated state. In this condition, dissolved sodium urate molecules no longer exist independently. Instead, driven by supersaturation, they spontaneously form oligomers through intermolecular hydrogen bonds and π-π stacking, subsequently assembling into nanoscale molecular aggregates. These aggregates (6) can further coalesce and dehydrate, evolving into AMSU (3, 7–9).

Current evidence regarding the physical form and biological relevance of AMSU primarily comes from *in vitro* crystallization models and ex vivo observations of patient-derived samples, rather than from real-time imaging within intact joints. Recently, Rodriguez-Navarro et al. identified an amorphous monosodium urate precursor deposited on the surface of collagen fibrils in pathological specimens from gout patients. This identification was achieved using a multimodal analytical approach that included Raman/FTIR spectroscopy, electron microscopy coupled with selected-area electron diffraction (SAED), and elemental analysis. Based on these findings, they proposed that the pathological formation of MSU in humans may follow an “AMSU precursor–nonclassical crystallization” pathway (3). This work provides critical morphological evidence supporting the existence of AMSU as a precursor phase of MSU at biologically relevant interfaces in humans; however, it should be noted that this remains a direct observation at the ex vivo level.

As a relatively stable and low-inflammatory precursor phase, AMSU tends to induce heterogeneous nucleation and deposition on damaged articular cartilage surfaces, synovial connective tissue, or cell-derived vesicles at various biological interfaces, and may participate in the uric acid crystallization kinetics process within a certain timescale (3, 10–14). This phenomenon explains the occurrence of several years of asymptomatic hyperuricemia. It remains to be determined whether AMSU can stably persist *in vivo* over extended periods and form a true biological “reservoir”. Future validation will require higher-resolution *in situ* imaging techniques and longitudinal studies. While the characteristics discussed above may provide a plausible mechanistic explanation for the prolonged asymptomatic phase observed in clinical hyperuricemia, they should not yet be interpreted as established *in vivo* evidence.

Under the influence of local pH fluctuations, changes in ionic strength, mechanical stress, or other microenvironmental factors, AMSU undergoes slow molecular rearrangement and structural ordering, leading to a “solid phase transformation” into MSU crystals, which have more stable thermodynamics and a regular lattice structure (15, 16).

**Figure 1:**
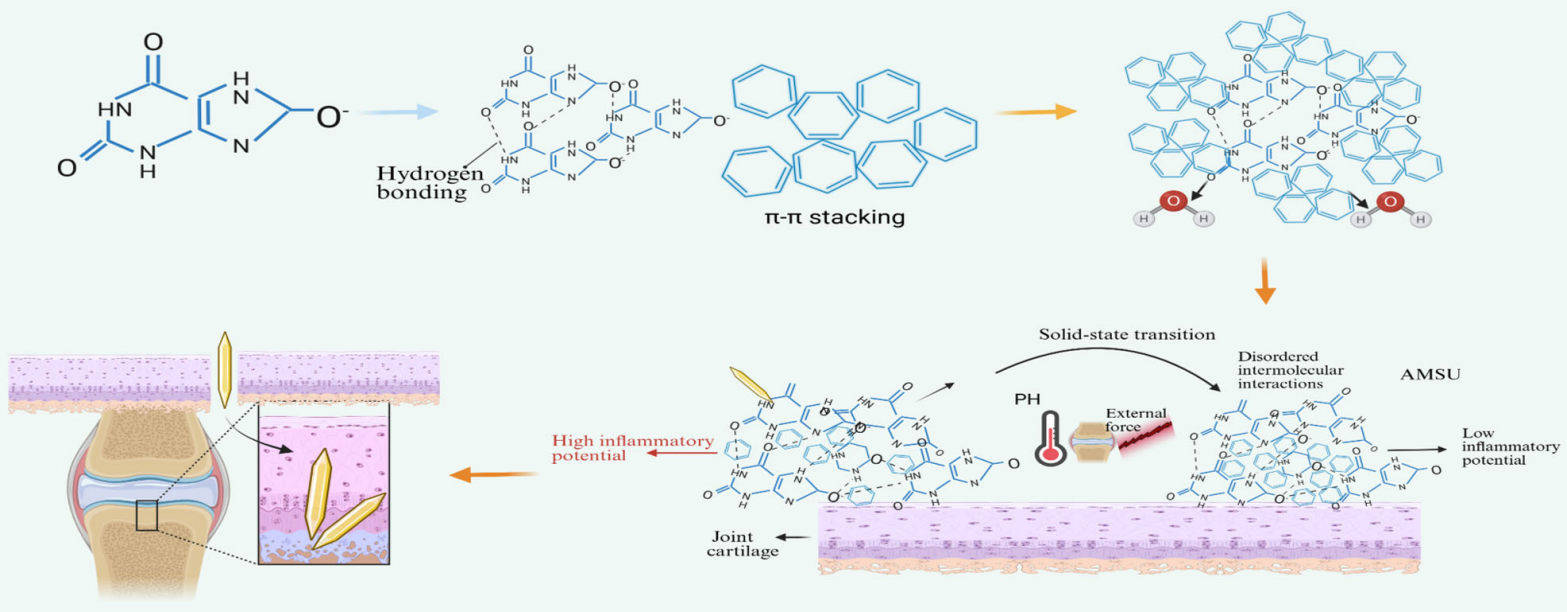
Formation of MSU crystals. High concentrations of SUA aggregate from monomers to oligomers under sustained supersaturation conditions, further forming AMSU, and then undergoing a solid-phase transition to produce needle-like MSUs.

After entering the synovial fluid environment, MSU crystals can nonspecifically adsorb proteins from synovial fluid and plasma. This process results in the formation of a protein coating, also known as an opsonization layer, on their surface. *In vitro* studies have demonstrated that MSU crystals can bind various humoral proteins, including serum proteins like γ-globulins and albumin, as well as interact with complement proteins and components of the contact system (see **Figure 1**).

This adsorption process does not seem to create a single, uniform, and static “membrane-like” structure. Instead, it is likely to form a complex, environment-dependent protein adsorption interface. The composition and relative abundance of this interface are affected by local protein availability, ionic conditions, and temporal factors, and it may undergo dynamic remodeling in both spatial and temporal dimensions (17, 18).

Therefore, while this phenomenon is frequently compared to a “protein corona,” there is currently insufficient direct evidence from systematic proteomic and time-resolved quantitative analyses that specifically characterizes the adsorbed layer on MSU crystal surfaces. A more accurate description at this time would be to view it as an “immunologically active interface dominated by protein adsorption and opsonization,” rather than as a fully defined classical protein corona structure (19–21).

From a functional perspective, the protein adsorption interface significantly alters the immunological properties of MSU crystals. This transformation changes the crystals from relatively inert physical deposits into immunologically active particles that are more easily recognized by the innate immune system, thereby increasing their inflammatory potential. Mechanistic studies have shown that MSU crystals can activate the complement system, including the alternative and lectin-associated pathways, with some evidence indicating the involvement of the classical pathway as well. This activation results in C3 cleavage and the deposition of opsonins, such as C3b, on the crystal surface. Additionally, opsonic molecules like C1q can directly bind to MSU crystals, facilitating immune recognition. These events of complement-mediated opsonization enhance phagocytosis by macrophages and neutrophils through engagement with complement receptors, further amplifying downstream inflammatory cascades (22–24).

However, it is important to note that, despite substantial evidence supporting the role of complement opsonization in MSU-induced immune activation, systematic data on the quantitative composition, spatial organization, and temporal dynamics of specific lectins, immunoglobulins, and other opsonic proteins within the crystal-associated coating layer are still limited.

Importantly, the effects of different adsorbed proteins on crystal-induced inflammation are unlikely to be uniform. Alongside complement-dependent pro-phagocytic and pro-inflammatory opsonization mechanisms, certain high-abundance humoral proteins—such as albumin or specific lipoproteins/apolipoproteins—may have modulatory or inhibitory effects when they bind to the crystal surface. By occupying molecular sites and causing steric hindrance, these proteins can partially shield high-affinity immune recognition sites, thus limiting the accumulation of additional opsonins. Under specific conditions, this interaction may also reduce crystal-induced phagocytosis and inflammatory responses (25, 26). Studies have shown that apoB-containing lipoproteins can bind to MSU crystals and retain their ability to suppress neutrophil inflammatory responses, even after other crystal-associated proteins have been removed. This suggests that lipoprotein adsorption may provide immunomodulatory or inhibitory effects (27).

Certain pattern recognition molecules, such as C-reactive protein (CRP), demonstrate site-selective binding to MSU crystals. They tend to associate preferentially with crystal edges or specific crystal faces instead of creating a uniform surface coating. This behavior indicates localized high-affinity interactions and kinetic selectivity at the protein–crystal interface. These interactions may further impact complement activation, immune cell recruitment, and the amplification of inflammation (28). Nonetheless, the inhibitory or interface-modulating effects are primarily supported by *in vitro* experiments or model-based inferences, leaving their stability and physiological relevance *in vivo* yet to be established.

Existing studies consistently indicate that the protein adsorption/opsonization interface formed on MSU crystals within humoral environments is crucial in shaping immune recognition and inflammatory outcomes. However, a comprehensive characterization of this interface—including its quantitative proteomic composition, time-resolved dynamic remodeling, and structure–function relationships—remains insufficient. Future research that integrates quantitative proteomics, time-resolved interfacial tracking, and functional validation will be vital in delineating the specific pro- or anti-inflammatory roles of individual adsorbed proteins. This approach will also provide a more robust theoretical foundation for therapeutic strategies aimed at modulating crystal-associated protein interfaces.

Current studies have found that although AMSU shares a similar short-range chemical composition with MSU, it lacks a long-range ordered crystal structure. As a result, the inflammatory activity of AMSU is significantly lower than that of MSU crystals. This difference may stem from the amorphous structure of AMSU, which alters its interaction with cell surface pattern recognition receptors, such as those related to NLRP3 inflammasome activation. Consequently, this reduces the activation ability of macrophages and other immune cells, thereby weakening the inflammatory response (19, 29). These findings suggest that maintaining the amorphous state of MSU or preventing its transformation into crystals could be a potential anti-inflammatory strategy.

The formation of MSU is a dynamic, multi-step pathological process that is actively regulated by biological interfaces and cells. This understanding introduces a new perspective on gout intervention: treatment should not only focus on reducing blood uric acid levels but also on stabilizing AMSU to prevent crystallization. Additionally, designing competitive molecules to interfere with the formation of the “protein coating” or removing early deposits during the asymptomatic period under imaging monitoring could help achieve prospective prevention and precise management of the disease (30). However, these concepts are primarily at the stage of mechanistic investigation and exploratory research. Their clinical feasibility will ultimately depend on future studies that clearly define the *in vivo* behavior, stability, and biological fate of AMSU.

It is important to emphasize that even in the absence of crystal formation, SUA itself possesses physiological and multiple pathogenic mechanisms. Therefore, before discussing the innate immune inflammation driven by MSU, it is essential to examine the signaling characteristics as well as the pathogenic and protective effects of SUA in its amorphous state.

## 3 Immunometabolic effects of soluble uric acid

### 3.1 The physiological role of soluble uric acid in redox homeostasis and metabolic regulation

Within the physiological concentration range of 200-400 µM, SUA can act as an endogenous allosteric inhibitor of nicotinamide adenine dinucleotide (NAD+) hydrolase CD38. It noncompetitively inhibits CD38’s NAD+ hydrolase and cyclase activities, leading to the maintenance or increase of NAD+ levels in cells. Increased NAD+ can activate SIRT1, and the activated SIRT1 can deacetylate proteins, inhibiting NF-κB. This process reduces the overtranscription of pro-IL-1β and NLRP3, thereby addressing inflammation at its source. Furthermore, activated SIRT1 can initiate the autophagy program, enhancing the cell’s ability to clean up and adapt metabolically. Autophagosomes not only engulf bacteria but also actively encapsulate and degrade the activated NLRP3 inflammasome complex and excess pro-IL-1β (31–33). Thus, SUA serves as an “endogenous guardian” that helps maintain immune metabolic homeostasis. Moreover, the increase in SUA levels with age may represent a compensatory response to the natural depletion of NAD+, helping to preserve NAD+ function through CD38 inhibition. This indicates that SUA is closely linked to the pathophysiology of aging, neurodegeneration, and metabolic diseases (34–36).

In addition, physiological concentrations of SUA exhibit anti-inflammatory and protective effects on articular cartilage. This protection is achieved through the selective intervention of specific pro-inflammatory signals. SUA effectively inhibits the phosphorylation of extracellular signal-regulated kinase (ERK) induced by pro-inflammatory factors such as IL-1 β and TNF - α. Consequently, it inhibits the activity of the AP-1 transcription factor complex without affecting other key pathways activated by the same pro-inflammatory stimuli, including the p38 MAPK, JNK MAPK, and NF - κ B signaling pathways. AP-1 is a core transcription factor that regulates the gene expression of essential enzymes involved in cartilage degradation, such as matrix metalloproteinase-13. By inhibiting the ERK/AP-1 axis, SUA directly downregulates the synthesis of destructive enzymes like MMP-13 and counteracts the inhibitory effects of pro-inflammatory cytokines on the expression of type II collagen mRNA, thereby protecting the core structural components of the cartilage matrix. This protective effect has been confirmed through experiments with three-dimensional cultured chondrocytes and tissue blocks, demonstrating SUA’s ability to maintain the balance of anabolic and catabolic metabolism in cartilage (37–40).

Additionally, SUA serves as an endogenous negative regulator of the innate immune system. In the context of chronic hyperuricemia and renal dysfunction, SUA disrupts intracellular pH homeostasis and cytoskeleton dynamics, specifically impairing the activation, internalization, and membrane recycling of β 2 integrin (LFA-1/Mac-1) in neutrophils. As a result, neutrophil adhesion, endothelial migration, and phagocytosis are inhibited, although the formation of neutrophil extracellular traps remains unaffected. While this inhibition of neutrophil recruitment can limit tissue damage in aseptic inflammation models, it can also lead to acquired immune deficiency during infectious inflammation, thereby increasing infection susceptibility in patients with chronic kidney disease (41, 42). Thus, the immune regulatory role of SUA functions as a double-edged sword, with its effects—whether “inhibition” or “activation”—dependent on the inflammatory nature of the local microenvironment (aseptic or infectious), the intensity of stimulation, and the cell-specific metabolic pathways influenced by SUA. For hyperuricemic patients with chronic kidney disease and recurrent infections, reducing SUA levels may restore immune defense and lower infection risks. This study suggests that medications promoting uric acid excretion may more effectively reverse SUA-mediated immunosuppression compared to those that merely inhibit its production. In gout patients, rapid uric acid reduction could trigger acute attacks due to crystal dissolution or CD38 “de-inhibition,” indicating a need for caution in advocating uric acid reduction during acute phases. Conversely, rapid SUA reduction may be beneficial for patients with immunosuppressive chronic kidney disease. This highlights the necessity for individualized treatment approaches, though both strategies require further high-level verification.

### 3.2 The pathological effects of persistent hyperuricemia on metabolic inflammation (see **Figure 2**)

**Figure 2:**
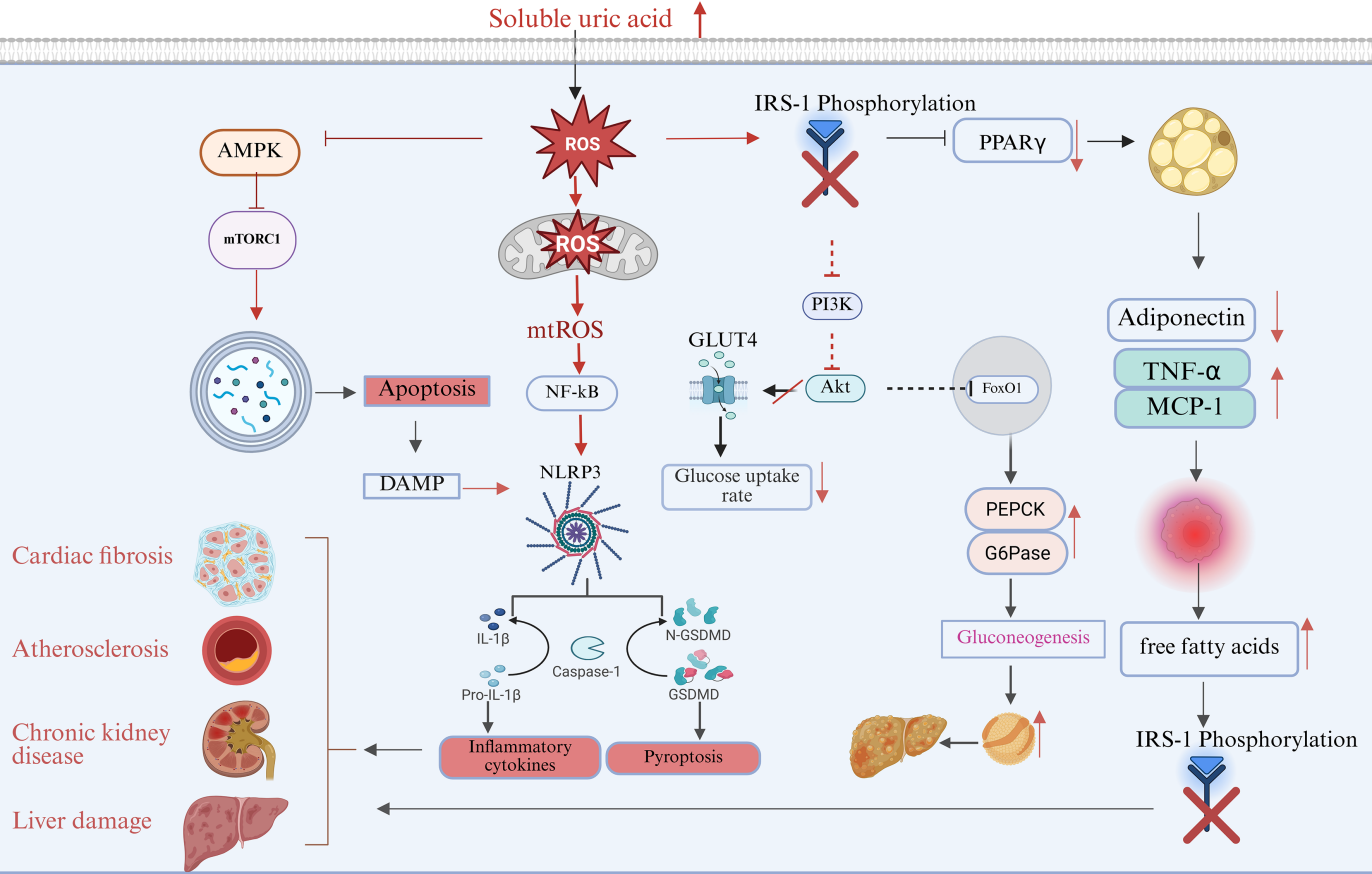
Schematic diagram of the multipathway pathogenesis of SUA. High concentrations of SUA promote tissue damage and chronic inflammation through multiple cellular and metabolic pathways. SUA induces ROS production and inhibits AMPK, leading to continuous activation of mTORC1, leading to autophagy inhibition and mitochondrial damage. mtROS further activates NF-κB and NLRP3 inflammasomes, promoting IL-1β/IL-18 release, pyroptosis, and increased inflammatory factors. SUA also inhibits IRS-1 phosphorylation, weakens the PI3K-Akt-GLUT4 pathway, and causes insulin resistance; The down-regulation of adiponectin and the increase of TNF-α/MCP-1 in adipose tissue aggravate lipolysis and hepatic gluconeogenesis, and the above abnormal signals jointly contribute to multi-organ lesions such as cardiac fibrosis, atherosclerosis, kidney injury, and liver metabolic disorders.

#### 3.2.1 SUA interferes with energy metabolism and causes insulin resistance

In the context of persistently elevated SUA levels, the homeostasis of intracellular energy metabolism is initially disrupted, with one of the primary targets being the crucial sensor of energy balance—AMP-activated protein kinase (AMPK) (43). Under physiological conditions, cellular energy depletion activates AMPK, which subsequently initiates catabolism to generate energy while inhibiting anabolism to conserve resources (44). However, high levels of SUA may amplify energy stress signaling. Concurrently, SUA-induced oxidative stress may impair full AMPK activation via post-translational modifications, leading to a condition in which activation cues persist but functional AMPK activity remains insufficient. This SUA-related functional disorder leads to the release of mTORC1 inhibition, which is a major regulator of anabolic metabolism. In an environment of energy stress, mTORC1 becomes continuously and abnormally active, driving unnecessary protein and lipid synthesis, which exacerbates the metabolic burden on cells. This results in blocked catabolism and impaired AMPK-dependent autophagy, leading to a failure in cellular autophagy processes (45, 46). Damaged mitochondria cannot be cleared in a timely manner and continue to release mtROS, creating a positive feedback loop of energy depletion and oxidative damage. Ultimately, this process induces cellular aging and apoptosis, while continuously releasing DAMPS and mtROS that activate the NLRP3 inflammasome, resulting in tissue-specific damage (47–49).

In addition to its indirect effects on energy metabolism through AMPK, SUA can directly impact mitochondria, the energy production centers of cells. SUA disrupts the electron transport chain within the mitochondrial inner membrane, leading to decreased electron transport efficiency and increased electron leakage. This results in reduced oxidative phosphorylation efficiency and energy output. Simultaneously, leaked electrons combine with oxygen molecules, causing bursts of mitochondrial-derived ROS, which have become a significant source of oxidative stress in cells (50, 51). In the context of energy perception disorders induced by SUA and mitochondrial dysfunction, when energy perception disorders and mitochondrial damage occur, abnormal energy metabolism and the resultant generation of reactive oxygen species activate a series of stress-sensitive signaling pathways, including the c-Jun N-terminal kinase, protein kinase C, and IKK β/NF-κB pathways (52, 53). These pathways share a common feature: they can phosphorylate a key protein in the insulin signaling pathway, insulin receptor substrate 1, at inappropriate serine/threonine sites. The serine phosphorylation of IRS-1 prevents its normal activation by the insulin receptor, hindering its binding to the downstream effector phosphatidylinositol 3 kinase. This results in severe inhibition of PI3K Akt pathway, which is the core axis of insulin signaling. Concurrently, metabolic disorders lead to an imbalance in lipid synthesis and degradation within cells, causing the accumulation of harmful lipid intermediates that result in lipotoxicity and further damage to insulin signaling (47, 54, 55).

#### 3.2.2 Abnormal energy metabolism of SUA and insulin resistance aggravate oxidative stress

Building on the previously discussed SUA-induced disturbances in energy metabolism and insulin resistance, the onset and exacerbation of oxidative stress represent a crucial pathological amplification mechanism.

First, under high metabolic stress related to SUA, dysfunctional mitochondria are considered a potential source of sustained ROS generation. The integrity of the electron transport chain is compromised, significantly increasing electron leakage from dysfunctional mitochondria. As a result, these mitochondria shift from being the “energy factory” of cells to becoming the primary pathological source of reactive oxygen species, such as superoxide anion (56, 57).

Second, SUA-induced insulin resistance state activates systemic ROS generation pathways. Hyperinsulinemia, which accompanies insulin resistance, along with early low-grade inflammatory signals, can further stimulate the expression and activation of NADPH oxidase (particularly the NOx subtype) on the cell membrane. This enzyme is induced to express and activate in considerable amounts, catalyzing the reduction of molecular oxygen to produce superoxide anion, thereby establishing a systematic mechanism for ROS production at the cell membrane level (58).

Third, In the context of persistent hyperuricemia-related metabolic disorders, the endogenous antioxidant defense system becomes weakened. The disorder in energy metabolism and the imbalance of redox homeostasis can lead to excessive depletion of crucial endogenous antioxidant substances, such as glutathione, superoxide dismutase, and thioredoxin. This also inhibits the biosynthesis of related antioxidant enzymes, resulting in a significant decline in the inherent ability of cells to scavenge free radicals. Consequently, an unbalanced state arises, characterized by “increased ROS production” and “insufficient ROS clearance,” available population-based and experimental studies suggest a tendency toward elevated intracellular oxidative stress (59–61).

#### 3.2.3 Oxidative stress triggers and amplifies the chronic inflammatory response

When SUA-induced metabolic stress and oxidative stress are sustained, the inflammatory response is consequently triggered and exacerbated. Excessive ROS serve not only as a direct agent causing damage to biological macromolecules but also as a potent second messenger that activates key pro-inflammatory signaling pathways, such as NF-κB and MAPK, through the oxidative modification of essential signaling proteins (62). Once activated, these transcription factors translocate to the nucleus, initiating the expression of various pro-inflammatory cytokines and chemokines, including TNF-α and IL-6. This response constitutes the “first wave” of the inflammatory signal and leads to the upregulation of transcriptional expression of NLRP3,pro-IL-1β and pro-IL-18, thereby preparing the molecular groundwork for inflammasome assembly.

Oxidative stress, coupled with mtROS and mtDNA released from mitochondrial dysfunction, creates a potent “danger signal” (48, 63, 64). Following the assembly of the inflammasome, caspase-1 is activated through self-cleavage. The activated caspase-1 then cleaves and activates the precursors of IL-1 β and IL-18, transforming them into their mature, biologically active forms, which are subsequently secreted outside the cell. Simultaneously, caspase-1 cleaves gasdermin D (GSDMD), triggering a form of inflammatory programmed cell death known as pyroptosis. This process results in the release of cellular contents and a multitude of inflammatory mediators. Consequently, TNF-α, IL-6, and other inflammatory factors mediated by NF - κB pathway, along with IL-1β, IL-18, and pyroptotic products specifically mediated by the NLRP3 inflammasome, create a synergistic amplification effect within the tissue microenvironment. IL-1 β and other cytokines further activate and recruit immune cells, enhancing NF-κB and other signaling pathways, thereby establishing a self-sustaining positive feedback inflammatory cycle (65–67). Driven by sustained metabolic and oxidative stress, inflammatory signaling may extend beyond intracellular abnormalities and progressively shape the surrounding tissue microenvironment, giving rise to a chronic, low-grade yet potentially amplifying state of “metabolic inflammation.” This persistent inflammatory milieu may create a permissive pathological context for subsequent structural remodeling and functional impairment of target organs.

### 3.3 Soluble uric acid’s organ-specific effects on metabolic dysfunction

#### 3.3.1 Soluble uric acid-induced endothelial dysfunction and cardiovascular remodeling (**Figure 3**)

**Figure 3:**
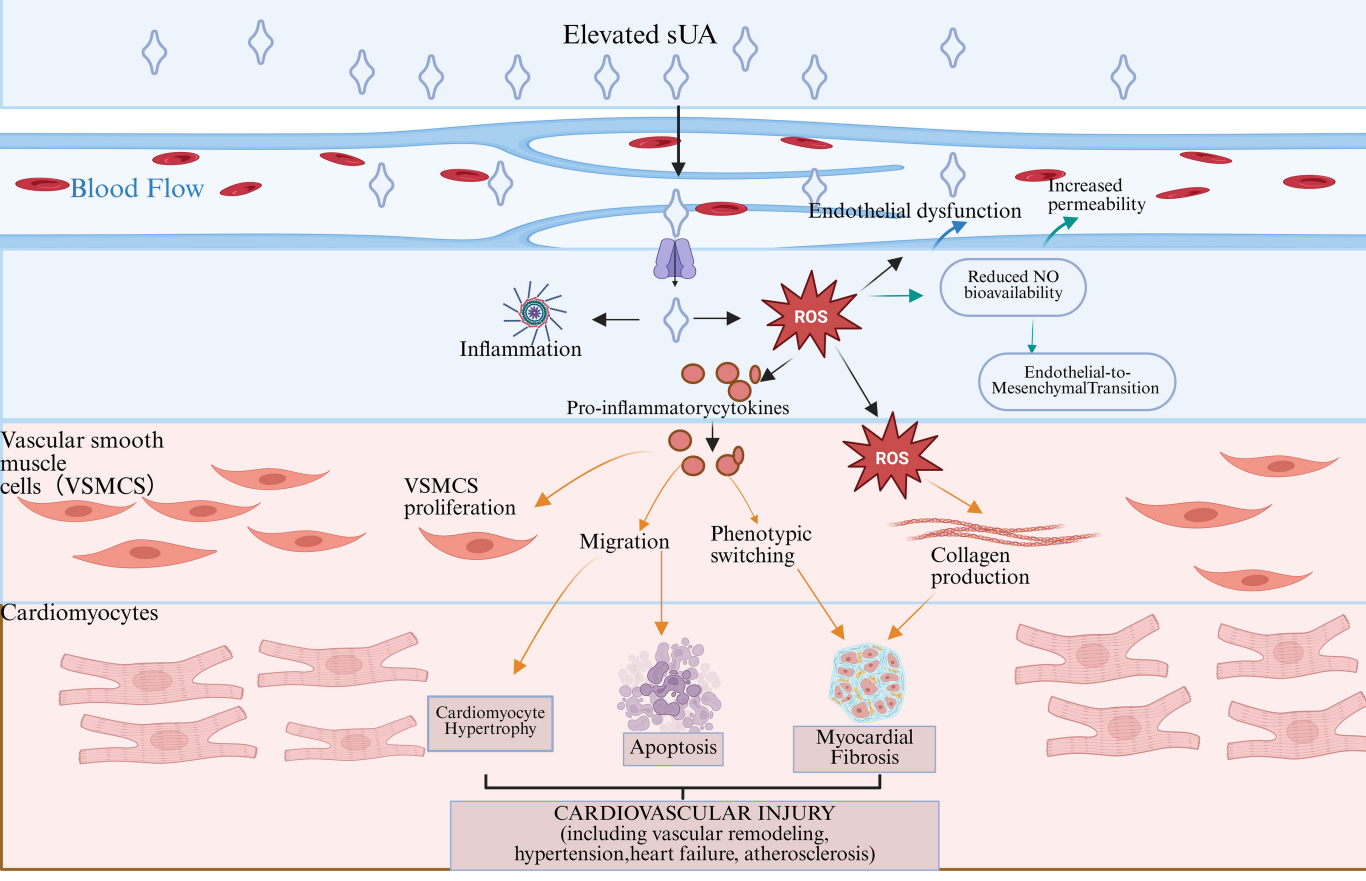
Pathological mechanisms linking SUA to cardiovascular injury. Elevated soluble uric acid promotes endothelial dysfunction through oxidative stress and inflammatory activation, leading to impaired nitric oxide signaling and increased vascular permeability. These changes drive vascular smooth muscle cell remodeling and extracellular matrix accumulation, which in turn contribute to cardiomyocyte hypertrophy, apoptosis, and myocardial fibrosis. Together, SUA-induced vascular and myocardial alterations underpin the development of cardiovascular injury, including hypertension, atherosclerosis, and heart failure.

Persistently elevated SUA in the heart can act as a damage-associated molecular pattern under pathological conditions, recognized by Toll-like receptors (such as TLR4) on the surface of cardiomyocytes and immune cells. This recognition initiates the NF-κB–mediated transcription of inflammatory genes, referred to as the “first signal”. Concurrently, high SUA induces mitochondrial dysfunction, leading to a surge of mitochondrial reactive oxygen species (mtROS). This surge provides the “second signal” necessary for NLRP3 inflammasome assembly. The convergence of these two signals activates the NLRP3 inflammasome, which catalyzes the maturation and release of IL-1β and IL-18. This process persistently activates cardiac fibroblasts and promotes excessive extracellular matrix deposition, contributing to the development of progressive myocardial interstitial fibrosis. Such structural remodeling impairs ventricular diastolic compliance and disrupts the uniformity of electrical conduction, forming the pathological basis for heart failure with preserved ejection fraction (HFpEF) and cardiac arrhythmias (68–71).

In the endothelium of blood vessel walls, long-term high SUA-related ROS attacks significantly increase the expression of vascular cell adhesion molecule-1, intercellular adhesion molecule-1, and E-selectin on endothelial cells, enhancing lipid permeability. This process also damages the tight junctions between endothelial cells, increases vascular permeability, and reduces the expression of anticoagulant substances while elevating procoagulant substances, creating a procoagulant state. In this environment, the endothelium, characterized by high adhesion molecule expression, aggressively “captures” monocytes from the bloodstream, facilitating their adherence and migration to the endothelium. Low-density lipoprotein (LDL) that infiltrates and accumulates beneath the endothelium undergoes oxidation, transforming into oxidized low-density lipoprotein (ox LDL) due to local high levels of ROS. This oxLDL is then engulfed by macrophages derived from the migrating monocytes, ultimately forming the characteristic foam cells associated with atherosclerosis (72, 73). Sustained oxidative stress and inflammatory cytokine release driven by elevated SUA may upregulate and enhance the expression and activity of matrix metalloproteinases (MMPs) in vascular wall cells, particularly macrophages and vascular smooth muscle cells. MMPs mediate the degradation of collagen and other extracellular matrix components within the fibrous cap, thereby disturbing the dynamic balance of collagen synthesis and degradation that underlies plaque stability.When collagen degradation becomes relatively enhanced and compensatory synthesis—primarily mediated by vascular smooth muscle cells—is insufficient, the structural integrity of the fibrous cap may be compromised. This is characterized by reduced collagen content and diminished mechanical strength, contributing to increased plaque vulnerability.Under such conditions, plaque rupture or superficial erosion may expose thrombogenic components to the circulating blood, triggering platelet adhesion and activation as well as the coagulation cascade, leading to thrombus formation of varying extent. In some cases, thrombi may progress to hemodynamically significant stenosis or even occlusion, which is associated with an increased risk of acute myocardial infarction or ischemic stroke. (68, 74–77).

#### 3.3.2 Soluble uric acid-mediated renal metabolic stress and fibrosis progression (**Figure 4**)

**Figure 4:**
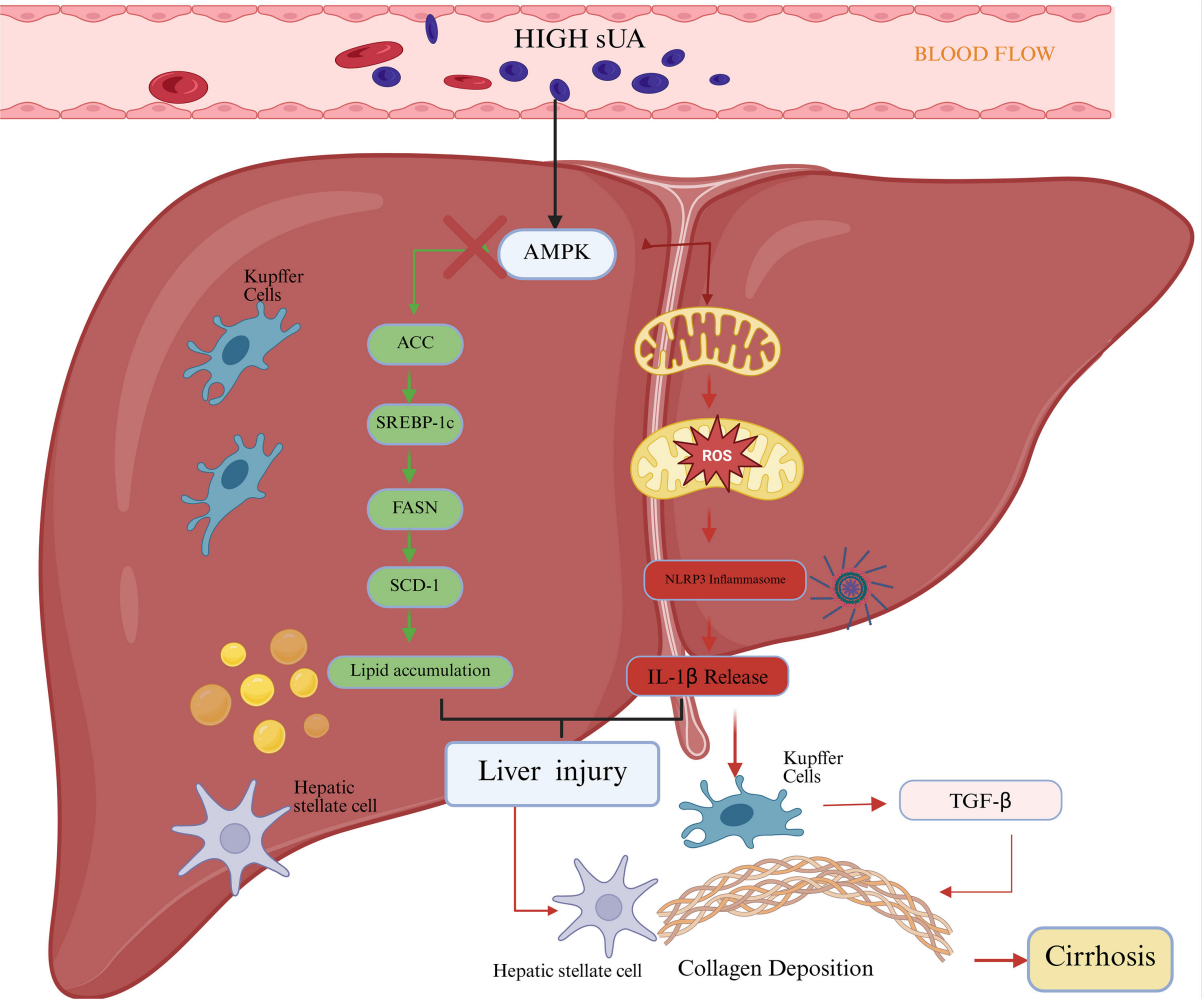
SUA induces liver injury and cirrhosis via AMPK-mediated lipid accumulation and inflammation. Elevated circulating SUA enters the liver and disrupts hepatic metabolic homeostasis. High SUA suppresses AMPK activity, thereby relieving inhibition of ACC and activating downstream lipogenic pathways, including SREBP-1c, fatty acid synthase (FASN), and stearoyl-CoA desaturase-1 (SCD-1), leading to excessive lipid accumulation in hepatocytes. Concurrently, SUA induces mitochondrial dysfunction and excessive mitochondrial ROS production, which together with lipid overload activates the NLRP3 inflammasome in hepatocytes and Kupffer cells. Inflammasome activation promotes IL-1β release, amplifying hepatic inflammation and hepatocellular injury. Pro-fibrotic mediators, particularly TGF-β, drive hepatic stellate cell activation and transdifferentiation into myofibroblasts, resulting in collagen deposition and progressive liver fibrosis, ultimately contributing to cirrhosis.

In the kidneys, metabolically active proximal tubular epithelial cells are particularly vulnerable to energy stress. SUA-induced oxidative damage and ATP depletion result in cell apoptosis and aging. SASP factors and DAMS create a chronic inflammatory microenvironment that continuously recruits and activates macrophages and NLRP3 inflammasomes (78, 79). The cleavage of gsdmd mediated by caspase-1 triggers pyroptosis. The DAMPs released by dying cells further amplify the inflammatory signal, driving myofibroblast activation and collagen deposition, which culminates in tubulointerstitial fibrosis. As fibrosis progresses, nephrons are replaced by scar tissue, leading to a gradual decline in renal function, which represents the core pathological process underlying the development of chronic kidney disease (CKD) (80, 81).

#### 3.3.3 The role of soluble uric acid in liver lipid metabolism and inflammatory injury (**Figure 5**)

**Figure 5:**
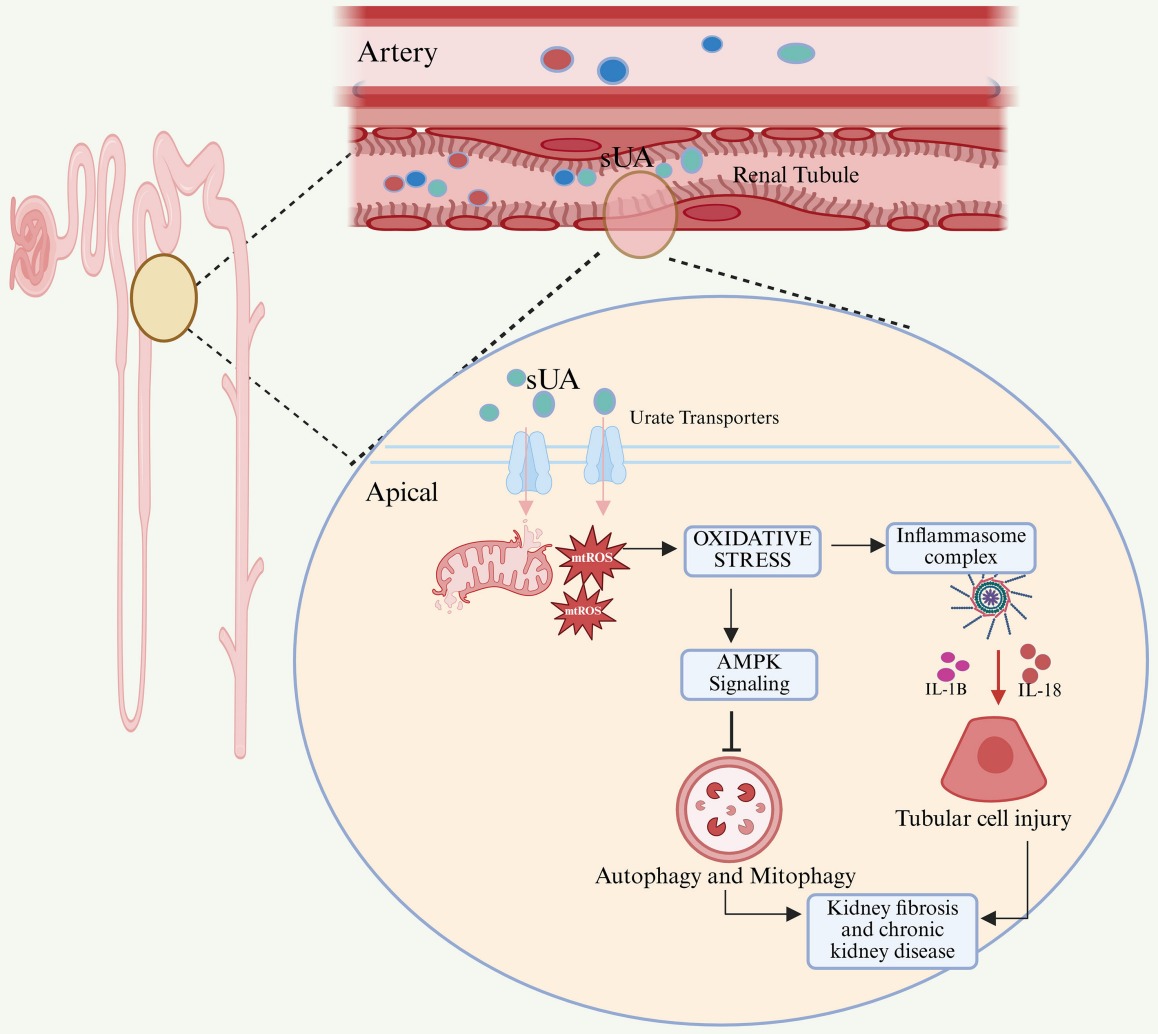
Pathological mechanisms of SUA on kidney damage. Circulating soluble uric acid is transported into renal tubular epithelial cells through apical urate transporters. Intracellular accumulation of SUA promotes mitochondrial ROS production, leading to oxidative stress. Oxidative stress subsequently activates the inflammasome, resulting in increased secretion of pro-inflammatory cytokines IL-1β and IL-18 and causing tubular cell injury. In parallel, oxidative stress suppresses AMPK signaling, thereby impairing autophagy and mitophagy. Dysregulation of these protective pathways contributes to kidney fibrosis and the progression of chronic kidney disease.

In the liver, persistently elevated SUA under pathological conditions may disturb hepatocellular lipid homeostasis by interfering with energy-sensing pathways and autophagic clearance mechanisms. Under physiological conditions, AMPK regulates key metabolic nodes, including acetyl-CoA carboxylase (ACC), through phosphorylation, thereby suppressing *de novo* fatty acid synthesis and promoting lipid oxidation. In contrast, under high-SUA–associated stress conditions, available evidence suggests that AMPK signaling may be suppressed or functionally insufficient, shifting ACC-mediated regulation toward a more lipogenic state and contributing to hepatic lipid accumulation.Concurrently, impaired autophagic flux,particularly dysregulated lipophagy,may reduce the efficiency of lipid droplet degradation and clearance, thereby further exacerbating intracellular lipid overload. (82, 83). In the context of metabolic disequilibrium, excessive lipid accumulation and mtROS activate the NLRP3 inflammasome in both hepatocytes and Kupffer cells. This activation is mechanistically linked to pathological changes, including hepatocellular ballooning, cellular injury, and necrosis. As the inflammatory response unfolds, the release of the profibrotic cytokine transforming growth factor-β (TGF-β) promotes the transdifferentiation of hepatic stellate cells into a myofibroblast phenotype. This process leads to excessive deposition of collagen and extracellular matrix, therefore, AMPK–ACC dysregulation and inflammation–fibrosis signaling linked to elevated SUA may constitute one of several mechanisms implicated in the progression from NAFLD to NASH and cirrhosis (50, 84).

#### 3.3.4 Overall imbalance of the metabolic organ network

SUA-induced inflammation and insulin resistance create a destructive network in key metabolic organs. In the liver, the inhibition of the PI3K/Akt axis increases the activity of glycogen synthase kinase 3 (GSK3), thereby blocking glycogen synthesis. Concurrently, the inhibitory effect of insulin on the transcription factor FoxO1 is diminished, leading to up-regulation of key gluconeogenesis enzymes (PEPCK and G6Pase). This results in hypergluconeogenesis and fasting hyperglycemia (85, 86). At the level of lipid metabolism, insulin resistance diminishes the normal inhibition of mitochondrial fatty acid oxidation, promoting the abnormal synthesis of very low-density lipoprotein and the accumulation of lipid droplets in the liver. This forms the molecular basis for metabolism-related fatty liver disease (87, 88).

In skeletal muscle, decreased Akt activity prevents the glucose transporter GLUT4 from translocating from intracellular vesicles to the cell membrane, significantly reducing glucose uptake (89, 90). At the same time, SUA and ROS can directly damage the mitochondrial electron transport chain complex, decreasing the coupling efficiency of oxidative phosphorylation and ATP synthesis (91). Additionally, the expression of peroxisome proliferator-activated receptor γ coactivator 1 α (PGC-1 α) is down-regulated, which weakens mitochondrial biogenesis and autophagy. This leads to mitochondrial dysfunction, a shift in cell energy utilization from high-efficiency oxidation to low-efficiency glycolysis, reduced basic energy consumption, and increased susceptibility to obesity (92). The reduced ability of muscle to oxidize fatty acids allows free fatty acids (FFA) to return to the liver, further promoting liver steatosis and exacerbating systemic lipotoxicity (93).

In adipose tissue, the uptake of SUA by adipocytes can disrupt insulin signaling, inhibit the activity of peroxisome proliferator-activated receptor γ (PPAR γ), and lead to disorders in preadipocyte differentiation disorder, mature adipocyte, as well as hypertrophy and dysfunction in mature adipocytes. This disruption results in an imbalance in adipokine secretion: levels of the insulin-sensitizing and anti-inflammatory factor adiponectin decrease significantly, while pro-inflammatory factors such as leptin, TNF - α and MCP-1 increase. This shift promotes macrophage infiltration and amplifies local chronic inflammation (94–96). Impaired insulin signaling further leads to the disinhibition of hormone-sensitive lipase, resulting in enhanced abnormal basal lipolysis and a continuous overflow of free fatty acids FFA. This “lipid overflow” occurs due to the failure of adipose tissue to store lipids, contributing to hyperlipidemia (97). Excessive FFA then spills into the liver, muscle, and pancreas, driving ectopic lipid deposition, aggravating insulin resistance, and damaging β-cell function. Consequently, adipose tissue becomes a persistent source of chronic low-grade inflammation, which further impairs systemic insulin sensitivity through circulating pro-inflammatory factors (98–100).

These interconnected processes exacerbate metabolic inflammation, leading to systemic metabolic disorders and forming a network of cellular interactions that contribute to SUA-mediated insulin resistance. In metabolic terms, this manifests as hyperinsulinemia, elevated fasting blood glucose, and impaired glucose tolerance. Organically, it is reflected in liver steatosis, muscle mitochondrial dysfunction, adipose tissue inflammation, and decreased energy expenditure. Systemically, it is closely associated with metabolic syndrome, type 2 diabetes, atherosclerosis, hypertension, and other comorbidities. Clinical studies have confirmed a significant positive correlation between serum uric acid levels and insulin resistance indices, indicating that uric acid-lowering therapies can improve insulin sensitivity to some extent. This suggests that SUA-driven metabolic and immune interactions play a critical role in the development of metabolic syndrome.

## 4 Pathogenic mechanism of sodium urate crystals (see **Figure 6**)

**Figure 6:**
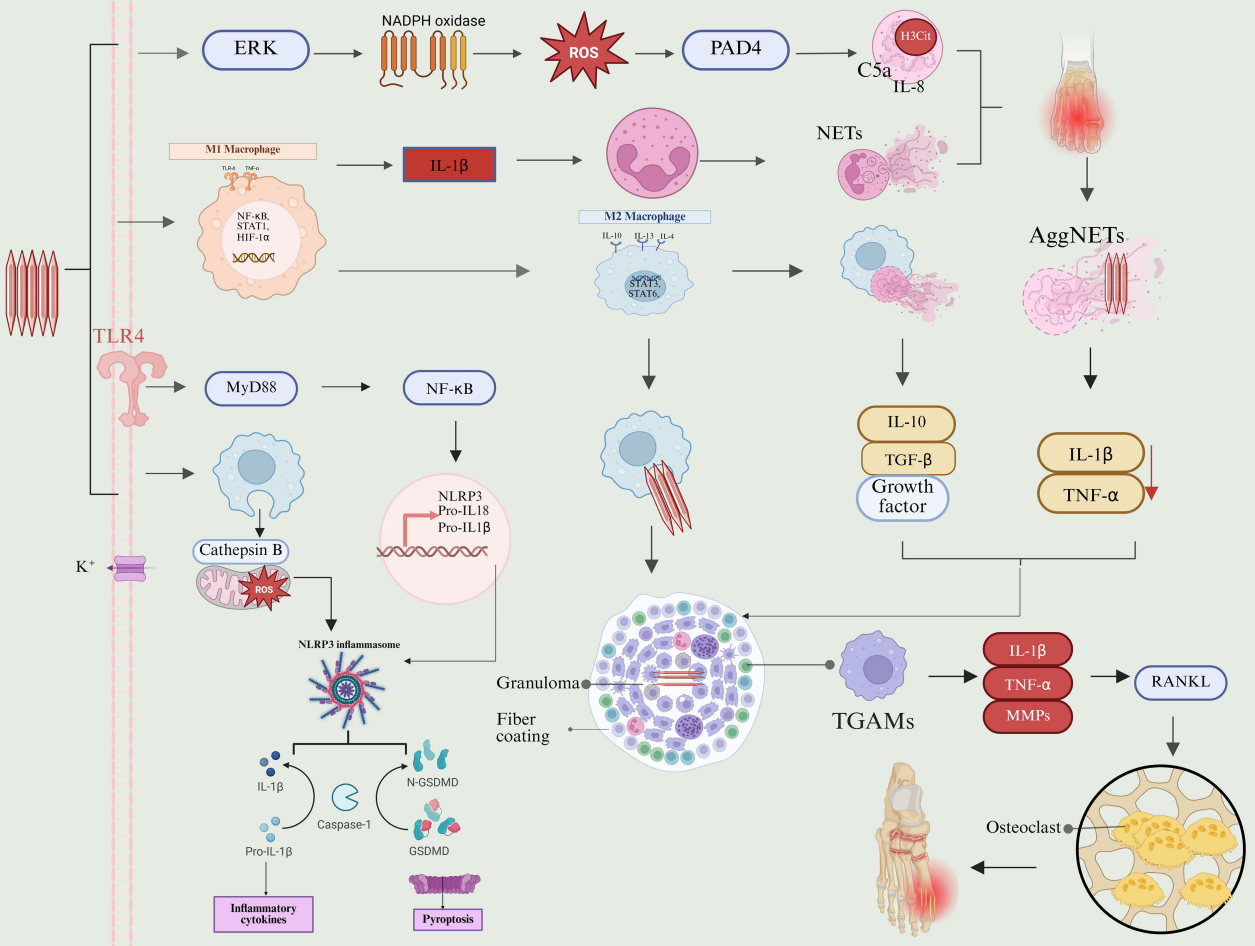
Pathogenic mechanisms of MSU. MSU crystals are engulfed by synovial macrophages after entering the joint, triggering lysosomal damage, K^+^ efflux and mtROS elevation, activating NLRP3 inflammasomes and releasing large amounts of IL-1β. IL-1β induces rapid recruitment of neutrophils and forms NETs, amplifying local inflammation. With the gradual clearance of crystals, macrophages shift from M1 to M2, secrete IL-10 and TGF-β to promote inflammation termination and tissue repair, and form tophi in the chronic stage, which is composed of three layers: MSU crystallization core, granuloma cell layer, and fibrous envelope.

When soluble uric acid undergoes prolonged heterogeneous nucleation and growth in a supersaturated physiological environment, it ultimately precipitates to form sodium urate crystals (3, 16). These crystals tend to deposit preferentially in areas with slow blood flow or low temperatures, such as peripheral facet joints, auricles, and renal papillae. The initial formation of these crystals is often asymptomatic; however, when exposed to immune cell environments or local tissue damage, they can induce typical sterile inflammation (101). The surface of MSU crystal carries a negative charge, enabling them to adsorb complement proteins, cytokines, and immunoglobulins. This transformation allows the crystals to act as dams that are recognized by the innate immune system, triggering a robust inflammatory response (102). This process signifies that gouty inflammation has transitioned from the physicochemical stage into the immune activation stage.

### 4.1 Initiation stage of inflammation: Intrinsic immune recognition of MSU and dual-signal inflammasome activation

The immune response induced by MSU follows the “double signal model.” First, toll-like receptors TLR2 and TLR4 recognize the crystal structure and recruit the intracellular adaptor protein myeloid differentiation primary response protein 88 (MyD88). This initiates the classical MyD88-dependent TLR signaling pathway, activating downstream Irak kinase and TRAF6, which then activates the TAK1-IKK signaling axis. The IKK complex phosphorylates and degrades the inhibitor protein IκB of NF-κB, allowing NF-κB to enter the nucleus. This activation of the NF-κB signaling pathway provides a pre-excitation signal for cells, upregulating pro-inflammatory cytokines TNF - α, IL-6 and chemokines (such as il-8/cxcl8). It also induces the transcriptional expression of NLRP3 inflammasome components and precursor interleukin-1 β (pro – IL-1 β), delivering the second signal for the innate immune response (103–105). This process primarily facilitates the transcriptional “priming signal” necessary for inflammasome activation and does not directly induce the secretion of mature cytokines. Concurrently, sodium urate crystals activate the bypass pathway and lectin pathway of the complement system, leading to the production of C5a, C3a, and other allergic toxins, as well as C3b and other opsonin fragments. C5a, one of the strongest chemokines, recruits a substantial number of neutrophils from the blood into the articular cavity. Both C5a and C3a enhance vascular permeability, resulting in plasma exudation and joint swelling. Additionally, C3b deposits on the crystal surface, conditioning it and enhancing the phagocytic and clearance abilities of macrophages (106).

Subsequently, when macrophages attempt to phagocytize MSU crystals, the sharp structure of the crystals causes lysosomal membrane rupture. This triggers a series of intracellular events, including cathepsin B release, K+ efflux, mitochondrial membrane potential decline, and ROS generation, which activate NLRP3 oligomerization. This process assembles mature inflammatory body complexes with the adaptor protein ASC and precursor caspase-1. Activated caspase-1 cleaves Pro – IL-1 β and pro – IL-18 to produce their mature forms, promoting the massive release of IL-1 β and IL-18, both of which exhibit strong inflammatory activity and trigger an acute inflammatory response (105, 107). IL-1 β further activates downstream signaling pathways, resulting in systemic and local reactions, such as fever, severe pain, significant vasodilation, and endothelial cell activation. In addition, IL-1 β can strongly upregulates the expression of vascular endothelial cell adhesion molecules and promotes the production of other chemokines, thereby amplifying the recruitment and infiltration of neutrophils and establishing a positive feedback cycle of inflammation (108).

At the same time, activated caspase-1 cleaved gasdermin D protein, triggering cell death. The N-terminal domain of gasdermin D, released after cleavage, exhibits membrane perforation activity and oligomerizes on the inner side of the cell membrane to form functional pores measuring approximately 10–20 nm. These channels disrupt the ionic homeostasis of cells, leading to water influx, cell osmotic swelling, and ultimately causing cell membrane rupture. This process releases a significant amount of intracellular mature alarm factors, such as IL-1 β, IL-18, high mobility group protein B1, and various damage-associated molecular patterns (DAMPs) into the extracellular space. These factors continuously stimulate the TLR and purinergic receptors (such as P2X7) of surrounding cells, creating a positive feedback loop that allows inflammation to amplify and trigger systemic acute inflammation (108, 109).

### 4.2 Acute inflammatory amplification phase: neutrophil recruitment, NETosis, and programmed cell death

The recruitment and activation of IL-1 β, TNF - α, complement fragment C5a, and chemical factors produced by macrophages and synoviocytes lead to a rapid influx of neutrophils into the articular cavity, marking the peak of the local inflammatory response, shows more significant performance under high crystal load conditions.

#### 4.2.1 NETosis as a major effector mechanism of acute inflammation

The unique form of programmed cell death exhibited by neutrophils in response to MSU crystals is known as netosis. This process is characterized by chromatin deconcentration, nuclear membrane rupture, and the formation of neutrophil extracellular traps (NETs) (110).

The core mechanism underlying netosis relies on ROS production mediated by NADPH oxidase. MSU crystals detect exogenous purine stimulation by activating purinergic receptors, which initiates the Raf/MEK/ERK signaling cascade. The phosphorylation of ERK facilitates the assembly and activation of NADPH oxidase (NOX2), leading to an oxidative burst. The ROS generated by NOX2 acts as both a signaling and effector molecule involved in NET formation. ROS promote the nuclear entry of azurol granulosa protein (NE, MPO) and activate PAD4, resulting in histone citrullination (h3cit) and chromatin de-aggregation. Subsequently, the nuclear membrane ruptures, and DNA is expelled, forming a net structure (111, 112). Concurrently, excessive ROS activate the necrotic signaling pathway involving RIPK3 and MLKL. RIPK3 promotes the phosphorylation of MLKL, leading to its insertion into the cell membrane and nuclear membrane, which creates membrane pores and accelerates chromatin leakage, thereby amplifying netosis (113–115).

#### 4.2.2 ROS-independent NETosis and inflammasome-related amplification loops

Besides the classic oxidative stress pathways, studies indicate that MSU crystals can also directly induce histone citrullination and NET formation through the TAK1/SYK/PAD4 axis or by directly activating the NF-κB pathway, thereby bypassing the classic ROS burst (110, 112). These ROS-independent pathways may become more prominent in chronic or low-grade inflammatory states. Together with the classical oxidative stress pathway, they help sustain the level of NETs in the inflammatory environment.

MSU crystals can activate the non-classical inflammasome pathway through TLR4 and other receptors. This activation leads to the activation of caspase-11, which cleaves GSDMD. The resulting GSDMD N-terminal fragment is responsible for membrane perforation. Additionally, GSDMD-N can specifically target the mitochondrial membrane, causing a collapse of the mitochondrial membrane potential and the release of mitochondrial DNA (mtDNA). As a potent damage-associated molecular pattern, the released mtDNA can further amplify the inflammatory signal and serve as a structural component of neutrophil extracellular traps (NETs), thus creating a self-amplifying loop of netosis (116, 117). When combined with the classical inflammasome pathway in the innate immune response, this process constructs a positive feedback inflammatory cycle that continuously activates inflammasome pathways.

#### 4.2.3 The dual role of NETs in inflammation self-limitation

Autophagy, as a key regulatory mechanism of inflammation, plays a complex role as a “bidirectional regulator” in MSU crystal-induced netosis (118, 119). In the early stages of netosis, MSU crystals can be upregulated by the Nfil3/Redd1 signaling axis or through the inhibition of Atg7, a key protein in autophagy. Activated Atg7 and the transcription factor p53 translocate to the nucleus, where they directly upregulate the expression of peptidyl arginine deiminase 4. PAD4 catalyzes the citrullination of the arginine residue on histone H3, facilitating chromatin depolymerization and creating conditions for the release of NETs. However, evidence also suggests that autophagy can have negative effects under certain conditions. Firstly, it can degrade key effector molecules of netosis and break down granular proteins such as neutrophil elastase through the autophagy-lysosome pathway, thereby weakening NET formation. Secondly, autophagy can promote the dissipation of inflammation and limit NET accumulation by enhancing the phagocytosis and clearance of formed NETs by macrophages, helping to prevent excessive tissue damage (119, 120).

In the late stages of an acute gout attack, a large number of NETs aggregate to form AggNETs (aggregated NETs). These dense aggregates not only serve as physical barriers but also play an active role in “inflammatory clearance” (121, 122). The reticular structure of AggNETs can physically capture MSU crystals, reducing their direct contact with immune cells. Additionally, AggNETs are rich in neutrophil serine protease, which can degrade pro-inflammatory factors such as IL-1 β and TNF - α, thereby attenuating the inflammatory cascade (123, 124). The formation of AggNETs signifies a shift in inflammation from amplification to resolution, providing a crucial cytological basis for the self-limitation of gout. In conclusion, NETs induced by MSU crystals exhibit dual pathological functions in gouty arthritis.

### 4.3 Resolution of inflammation and chronic remodeling phase: macrophage polarization and tissue remodeling

At the initial stage of MSU crystal deposition, microenvironment signals strongly drive macrophages toward M1 polarization. This process is characterized by a chain reaction termed “signal integration metabolic recombination effect amplification.” MSU crystals act as initial danger signals by binding to toll-like receptors and collaborating with cytokines such as interferon-γ and tumor necrosis factor-α. This interaction drives the M1 polarization program of macrophages through key signaling pathways, including NF-κB and JAK-STAT1. As HIF-1 α accumulates stably, the metabolic flow within macrophages rapidly shifts from mitochondrial oxidative phosphorylation (OXPHOS) to a high-throughput energy supply mode dominated by glycolysis (125). Glucose uptake increases due to the upregulation of GLUT1, while the regulation of key enzymes like HK2 and PKM2 alters the flow of glycolytic products. Notably, some PKM2 translocates into the nucleus, where it collaborates with HIF-1α to activate genes for the IL-1 β precursor and components of the NLRP3 inflammasome. Meanwhile, HK2 interacts with the mitochondrial membrane protein VDAC, disrupting mitochondrial membrane potential and promoting succinic acid accumulation and mtROS burst (126–128). These metabolic intermediates further enhance the stability of HIF-1α and activate NF-κB, creating a “metabolism signal resonance” loop. This process promotes the assembly of NLRP3 inflammasomes and caspase-1 activation, ultimately leading to cell death and the release of large amounts of mature IL-1β, resulting in neutrophil aggregation and acute inflammatory diffusion (129, 130). At this stage, the tissue microenvironment is characterized by acidification, hypoxia, and high lactate levels, creating a typical state of metabolic inflammatory amplification.

As acute inflammation gradually subsides, microenvironment signals shift to extinction signals such as IL-4, IL-13, IL-10, and the release of apoptotic neutrophils. These signals reshape transcription through the JAK-STAT6/STAT3 pathway and induce macrophages to transition into the M2 phenotype. At this point, energy metabolism is reprogrammed: glycolysis is inhibited, fatty acid β-oxidation (FAO) and oxidative phosphorylation regain dominance, the AMPK-mTOR axis is rebalanced, and mitochondrial structure and function are repaired (131–133).

This metabolic remodeling not only inhibits the pro-inflammatory process at the energy supply level but also directly supports the repair functions of M2 macrophages. Enhanced OXPHOS and NAD + metabolism enable cells to generate sufficient ATP to support efferocytosis, effectively recognizing and removing apoptotic neutrophils and tissue fragments. Phagocytosis further stimulates the secretion of IL-10, TGF - β, and growth factors, initiating a positive feedback cycle that promotes anti-inflammatory responses and tissue repair. This process facilitates the differentiation of synovitis and tissue reconstruction (134–136).

However, when the acute inflammatory response is self-limited through NETs and AGGNets, residual immune stimulation can cause inflammation to transition from a reversible acute phase to a chronic remodeling phase if the crystals are not completely removed (137). At this stage, the disruption of macrophage polarization balance and fibroblast activation work together, pushing the tissue toward a turning point of “inflammation self-limiting fibrosis immobilization.” This marks the evolution of gout lesions from a functional inflammatory response to the irreversible stage of structural reconstruction.

With the recurrence of the disease, long-term crystal load state not cleared, it progressed to the chronic stage, leading to changes in the immune response. Macrophages became the dominant cells, attempting to engulf the crystals; however, they failed because the crystals were resistant to lysosomal degradation. This failure resulted in the continuous activation of macrophages, which fused to form multinucleated giant cells. These giant cells, along with lymphocytes, clustered around the crystals, contributing to chronic granulomatous inflammation. Simultaneously, the body initiated a fibrosis repair process to isolate the lesions that could not be cleared. Recent single-cell multiomics studies have identified a unique class of “gout-associated macrophages” (tgams) in the granuloma area. These macrophages play a central role in intercellular communication and matrix remodeling by expressing osteopontin (SPP1) and other molecules (138). Activated fibroblasts secrete a significant amount of collagen fibers at the periphery of the granuloma, forming a dense capsule that ultimately creates the three-layer pathological structure of classic gout stones: the inner MSU crystal core, the middle chronic inflammatory granuloma zone, and the outer fibrous capsule. This structure is dynamic; the internal chronic inflammatory cells, particularly tgams, continue to release inflammatory mediators and proteases such as interleukin-1 β (IL-1 β), tumor necrosis factor - α (TNF - α), and matrix metalloproteinases (MMPs).

In addition to the space-occupying effect of the gout stone, the crystal masses deposited on the bone surface activate osteoclasts by upregulating the RANKL pathway. This activation results in “chisel-like” bone erosion and cystic changes with clear boundaries, ultimately leading to irreversible anatomical deformities and loss of joint function (139, 140).

Consequently, MSU crystals can shift local inflammation from a transient defense mechanism to ongoing tissue remodeling through the activation of inflammatory corpuscles and the programmed response of immune cells. This transformation becomes a key hub for uric acid-related inflammation, linking metabolic signals to tissue lesions.

## 5 Mechanism-oriented treatment strategies and frontier directions

### 5.1 Directly targeting SUA levels intervention strategies

Elevated serum uric acid is essential for the formation and deposition of MSU crystals. Consequently, the direct reduction of SUA levels is fundamental to the treatment of gout and hyperuricemia. Current interventions primarily focus on either inhibiting uric acid production or enhancing urate excretion. The main therapeutic benefit of these approaches comes from lowering the overall urate burden rather than from direct anti-inflammatory effects.

#### 5.1.1 Inhibition of uric acid production: xanthine oxidoreductase inhibitors

Xanthine oxidase is the key rate-limiting enzyme in uric acid production. XOR inhibitors reduce SUA levels by decreasing uric acid synthesis, making them one of the established strategies for lowering uric acid (141). Febuxostat, a selective non-purine XOR inhibitor, is safer for patients with impaired renal function. It significantly lowers blood uric acid levels and downregulates the expression of NLRP3 and IL-1 β, but these anti-inflammatory effects may be secondary consequences of reduced uric acid load, rather than their primary therapeutic target (142). Tigulostat, a new XOR inhibitor, has shown a dose-dependent reduction in uric acid during phase II/III clinical trials, with a curative effect comparable to existing medications and excellent tolerance (143).

#### 5.1.2 Promote uric acid excretion: URAT1 inhibitors

An abnormality in uric acid excretion constitutes a significant mechanism underlying hyperuricemia. Inhibitors of URAT1 decrease SUA levels by obstructing the reabsorption of uric acid within the proximal tubule, consequently enhancing uric acid excretion. Benzbromarone and probenecid inhibit URAT1-mediated reabsorption in the proximal convoluted tubules, thereby increasing uric acid excretion (144). Additionally, novel URAT1 selective inhibitors, such as lesinurad and dotinurad, offer higher selectivity and fewer drug interactions. These are often combined with XOR inhibitors to achieve a dual mechanism for uric acid reduction, its clinical benefits stem from the improvement of uric acid homeostasis (145).

### 5.2 Therapeutic strategies that indirectly affect SUA-related pathological processes through metabolic regulation

In addition to directly reducing uric acid levels, metabolic abnormalities may indirectly influence the onset and progression of gout by altering uric acid production, excretion, and susceptibility to inflammation. The primary therapeutic targets of relevant pharmacological agents do not encompass uric acid metabolism per se, and their effects on hyperuricemia are contingent upon specific contextual factors.

#### 5.2.1 AMPK activation and improvement of metabolic homeostasis: metformin

Metformin primarily exerts its pharmacological effects by activating AMPK and suppressing hepatic gluconeogenesis, thereby restoring glucose metabolic homeostasis. At the mechanistic level, AMPK activation may secondarily influence lipid metabolism, mitochondrial function, and inflammatory signaling pathways, potentially ameliorating pathological states associated with hyperuricemia. However, much of the evidence supporting these anti-inflammatory effects derives from mechanistic and preclinical studies, and their direct relevance in the setting of hyperuricemia-associated inflammation remains to be established through robust clinical investigation. (80, 146, 147). Accordingly, the anti-inflammatory benefits of metformin are more appropriately regarded as downstream or indirect consequences of metabolic modulation rather than its primary therapeutic target.

#### 5.2.2 Regulation of insulin resistance: pioglitazone

Pioglitazone, a classic PPAR γ agonist, improves insulin sensitivity and alleviates systemic lipotoxicity by promoting adipocyte differentiation, enhancing adiponectin secretion, and reducing the expression of TNF - α and MCP-1 (148). Animal and clinical studies indicate that pioglitazone not only enhances insulin resistance and glucose metabolism but also lowers serum uric acid levels, reduces endothelial dysfunction, and mitigates inflammatory responses, providing dual benefits for patients with hyperuricemia or metabolic syndrome (149, 150). However, these benefits are more plausibly interpreted as downstream consequences of improved metabolic and inflammatory milieus, rather than as effects directly targeting uric acid metabolism.In addition, these medications can cause adverse reactions such as water and sodium retention and weight gain, necessitating careful consideration of the patient’s cardiac function status.

### 5.3 Direct targeting of MSU crystals to induce inflammatory pathway intervention strategies

The acute inflammatory response triggered by MSU crystals primarily involves the activation of the NLRP3 inflammasome and the subsequent release of IL-1β. This pathway is therefore a crucial target for anti-inflammatory treatments in gout.

#### 5.3.1 Non-specific anti-inflammatory treatment: NSAIDs and colchicine

Traditional treatments like non-steroidal anti-inflammatory drugs (NSAIDs) and Colchicine can inhibit upstream signal amplification; however, their mechanisms of action do not directly target uric acid or MSU crystals. NSAIDs work by reducing the production of prostaglandin E2 and thromboxane through the inhibition of cyclooxygenase (COX-1/2), which in turn decreases vascular permeability and the pain response induced by inflammatory mediators (151). Colchicine, a classical microtubule inhibitor, binds to tubulin and blocks microtubule polymerization. This action inhibits chemotaxis, phagocytosis, and degranulation of neutrophils, effectively blocking the amplification of inflammation caused by lens and reducing the release of IL-1 β. By inhibiting the signaling axis of the P2X7 receptor and the NLRP3 inflammasome, colchicine can terminate the inflammatory response at the intracellular level. The inhibition of the P2X7–NLRP3–IL-1β axis should be viewed as a consequence of regulating the cytoskeleton and signal transduction, rather than as a direct inhibition of inflammasome assembly (152, 153). Accordingly, within the conceptual framework of this review, colchicine is best characterized as an anti-inflammatory modulator.

#### 5.3.2 Targeted IL-1β and NLRP3 inflammasome therapy

In cases of refractory or recurrent gout, biological agents targeting IL-1 β—the final effector molecule of the NLRP3 inflammasome—have demonstrated significant efficacy. Canakinumab an Firsekibart, a humanized IL-1 β monoclonal antibody, specifically binds to IL-1 β, blocking its interaction with receptors and thereby inhibiting IL-1 β mediated inflammatory signals. Anakinra, an IL-1 receptor antagonist, competitively inhibits the binding of IL-1 α/β to IL-1R1, providing rapid control of the inflammatory response. This makes it an important option for patients with refractory or frequently recurrent gout, although it still represents a downstream intervention (154, 155). Recently, a series of small molecule drugs that directly inhibit the assembly of the NLRP3 complex have been developed. Mcc950 (cp-456773) is the first compound confirmed to have a highly selective inhibitory effect, as it can directly block ATP hydrolysis and the conformational change of NLRP3, thus inhibiting its activation at the source (156). Meanwhile, dapansutrile (olt1177) administered orally, has shown good safety and an anti-inflammatory trend, although it has not yet been approved for the treatment of gout (157). Arhalofenate exerts dual urate-lowering and anti-inflammatory effects in gout by targeting both metabolic and inflammatory pathways. Mechanistically, it activates AMPK signaling in macrophages to suppress MSU crystal-induced inflammatory responses, while inhibiting renal urate reabsorption transporters to reduce serum urate levels, thereby decreasing gout flare risk and acute inflammation (158). In summary, the treatment of gout is evolving from traditional “broad-spectrum anti-inflammatory” approaches to a new phase of “precise inhibition of inflammatory pathways.” Future treatment for gout will likely shift from merely controlling symptoms to addressing the underlying pathological intervention.

### 5.4 Supportive interventions for gout stones and crystal load

Urine pH plays a crucial role in the development of sodium urate crystals. Alkalization treatment can disrupt the pathological cycle of “urate deposition, local inflammation, and kidney injury” by enhancing the solubility of uric acid and reducing crystal formation, crystal intervention strategies at the physicochemical level. Commonly used medications for this purpose include sodium bicarbonate and sodium potassium citrate. Recent studies have shown that urine alkalization not only creates a more favorable environment for urate dissolution but also indirectly reduces inflammation levels by inhibiting the activation of Toll like receptor (TLR) signaling, thereby offering renal protection (159, 160). For refractory gout, recombinant uricase can catalyze the oxidation of uric acid into the more soluble allantoin, facilitating the rapid removal of uric acid deposits and gout stones, and alleviating the crystal load. However, since allantoin is an exogenous protein, it can easily trigger antibody production, which may diminish its therapeutic effect. Consequently, its clinical use is generally limited to short-term or refractory cases (161).

### 5.5 Prospective intervention in crystalline biomineralization

From a pathological perspective, blocking MSU crystal formation and the upstream processes that enhance their immunogenicity represents a proactive intervention strategy focused on regulating crystallization and biomineralization. Instead of directly targeting established inflammatory responses, this approach seeks to prevent the formation of pro-inflammatory crystals at their source by modulating crystallization pathways and the properties of crystal interfaces. Currently, this field primarily comprises *in vitro* studies and conceptual proof-of-principle, lacking mature clinical therapeutic modalities, but it offers new mechanistic insights for gout research.

Two potentially viable pathways have been proposed. The first involves modulating the interactions between proteins and monosodium urate molecules or crystal interfaces to disrupt crystallization dynamics and alter crystal surface properties. The second pathway utilizes physical fields, such as ultrasound, to induce crystal de-structuring and disaggregation, thereby shifting pro-inflammatory MSU back toward low-immunogenic amorphous or precursor states.

At the molecular level, several research groups have designed peptides composed of arginine and tyrosine residues in varying sequences and lengths, based on key interaction sites between proteins and MSU crystals. These peptides were shown to inhibit crystal growth at low concentrations, providing a proof of concept for the development of novel therapeutic molecules that mimic the function of endogenous crystallization inhibitors and selectively target late-stage crystal growth (30, 162). It is important to note that studies of this type are primarily limited to *in vitro* experimental settings. As a result, their effects on crystal immunogenicity, inflammatory activation thresholds, and *in vivo* stability have not been systematically validated. Thus, these approaches should be viewed as conceptual molecular tools for targeting late-stage regulation of crystallization, rather than as direct therapeutic candidates.

In terms of physical therapy, studies indicate that external ultrasonic fields can disrupt the long-range ordered structure of MSU crystals through mechanical and cavitation effects. This induces a transformation to an amorphous state, promoting dissolution and offering a theoretical foundation for non-invasive dissolution of intra-articular deposits. However, it is important to note that ultrasound may also cause crystal fragmentation, producing smaller particles that could increase the risk of local inflammation in the short term. Clinical applications must carefully balance the benefits of dissolution efficiency against the potential for inflammation (6). However, the existing evidence is predominantly derived from *in vitro* models or animal studies; consequently, the safety window, dosing parameters, and long-term effects of this approach in clinical applications necessitate further systematic evaluation. The advancements in dual-energy CT and ultrasonic microscopy further enable the viSUAlization of microcrystalline deposits during asymptomatic periods (163). This capability provides an objective basis for initiating anti-uric acid or anti-crystallization therapy at the “preclinical stage,” facilitating primary prevention. However, the extent to which such observations warrant the initiation of early intervention, as well as the efficacy of these interventions in altering the natural history of the disease, remains unsupported by prospective clinical evidence.

In summary, strategies encompassing protein-mediated regulation of crystallization and crystal–interface properties, alongside ultrasound-induced crystal de-structuring, represent a “dual-pathway approach” designed to modulate the immunogenicity of monosodium urate crystals. This conceptual framework not only suggests novel avenues for disrupting the disease progression of early gout but also establishes a theoretical basis for the future evaluation of non-pharmacological or adjunctive interventions rooted in crystal morphology and kinetic dynamics. Nevertheless, the clinical translational potential of these methodologies requires further validation (See **Table 1**).

**Table 1:** Therapeutic interventions targeting SUA and MSU–driven mechanisms in gout.

Pathway/target	Mechanism of action	Representative agents or strategies	Clinical/developmental status	Predominant disease stage	Key considerations
Xanthine oxidoreductase	Suppression of uric acid production via inhibition of purine metabolism	Allopurinol, Febuxostat	Approved	SUA-dominant; mixed stage	Long-term serum urate target <6 mg/dL (lower in tophaceous or high crystal-burden disease); febuxostat use warrants consideration of cardiovascular safety signals
Dual urate–inflammatory axis (URAT1 + AMPK)	Dual urate-lowering and anti-inflammatory effects via inhibition of renal urate reabsorption and macrophage AMPK activation	Arhalofenate	Phase II	Mixed stage; high flare-risk patients	Represents a dual-acting strategy integrating urate lowering with direct anti-inflammatory effects; may reduce flare risk during urate-lowering initiation
Uricase replacement	Enzymatic oxidation of uric acid to the more soluble allantoin	Pegloticase	Approved (refractory gout)	MSU-dominant; mixed stage	Clinical use limited by immunogenicity, including anti-drug antibody development and infusion reactions
URAT1/OAT4/OAT10 (renal tubular transporters)	Inhibition of renal tubular urate reabsorption, enhancing uricosuria	Dotinurad, Benzbromarone, Probenecid	Approved	SUA-dominant; mixed stage	Benzbromarone is associated with hepatotoxicity; assessment of nephrolithiasis risk is recommended
NLRP3 inflammasome	Direct inhibition of NLRP3 inflammasome activation and assembly	Dapansutrile (OLT1177), MCC950, DFV890 (investigational)	Phase II/III (dapansutrile); early-stage development for others	MSU-dominant; mixed stage (flare modulation)	Represents a targeted anti-inflammatory strategy; no NLRP3 inhibitor is currently approved for gout
IL-1β signaling axis	Blockade of IL-1β–mediated inflammatory signaling	Canakinumab, Anakinra, Rilonacept,Firsekibart	Approved for selected indications or regions;Firsekibart: Phase III	MSU-dominant (acute or refractory flares)	Rapid suppression of inflammation; high cost and increased infection risk require careful patient selection
Microtubule dynamics/cytoskeleton	Inhibition of neutrophil migration and inflammasome-related signaling	Colchicine	Approved	MSU-dominant (acute flares and flare prophylaxis)	Low-dose regimens are preferred; renal and hepatic function, as well as drug–drug interactions, should be considered
Crystal–protein interface modulation	Regulation protein-mediated crystallization and interface	Arginine-/tyrosine-based peptides and related constructs (preclinical)	Proof-of-concept	Transitional (aMSU → MSU); mixed stage	An emerging “active crystal inhibition” approach aiming to modulate crystal bioactivity rather than systemic urate levels
Physical crystal disruption	Mechanical disruption of MSU crystal structure and deposits	Ultrasound- or cavitation-based crystal disruption (exploratory)	Conceptual/preclinical exploration	MSU-dominant (localized crystal burden)	Image-guided approaches may enable rapid crystal debulking; potential risks include secondary inflammation and crystal redistribution
Local microenvironment modulation	Improvement of urate solubility through urinary alkalinization	Potassium sodium hydrogen citrate	Approved	SUA-dominant; preventive stage	Typically used as an adjunctive strategy to enhance the efficacy of urate-lowering therapy

## 6 Summary and discussion

In summary, accumulating evidence indicates that uric acid is no longer viewed merely as a metabolic end product, but rather as a context-dependent modulator of metabolic regulation, redox homeostasis, and inflammatory signaling. Clinical and translational studies have firmly established that MSU crystals trigger acute gout flares through activation of the NLRP3 inflammasome. Moreover, persistent hyperuricemia is consistently associated with metabolic dysfunction and increased cardiovascular risk. However, the signaling functions of SUA in its non-crystalline state, as well as its causal contribution to complex clinical phenotypes at the population level, remain supported primarily by mechanistic and associative evidence, and require further integrative clinical validation.

At the mechanistic level, several metabolic–immune regulatory pathways have been proposed to participate in uric acid–related pathological processes. For instance, the SUA–CD38–NAD^+^–SIRT1 axis has been suggested as a potential regulatory node linking energy metabolism and inflammatory balance. Nonetheless, systematic population-based data are lacking to determine whether this axis exhibits nonlinear or bidirectional regulatory characteristics across different ages, metabolic backgrounds, and redox states. Similarly, current evidence regarding the preclinical regulation of MSU formation—such as the proposed “buffering” or stabilizing role of AMSU prior to crystal maturation—largely derives from *in vitro* models or indirect inference. These mechanisms should therefore be regarded as emerging research directions rather than established physiological paradigms.

Consistent clinical observations indicate that rapid urate lowering may precipitate acute gout flares; however, the underlying molecular and immunological mechanisms remain incompletely defined. Based on current understanding of pathophysiology and crystal dynamics, several plausible explanatory frameworks can be proposed. First, a rapid decline in serum urate levels may disrupt pre-existing crystal–dissolution equilibria, leading to structural rearrangement of deposited MSU crystals, exposure of previously concealed crystal surfaces, or mobilization of microcrystalline particles. These changes may enhance phagocytic recognition and inflammasome activation. Second, given that uric acid contributes to systemic antioxidant capacity, abrupt reductions in its concentration may transiently alter local redox balance, potentially lowering the threshold for inflammatory activation or reshaping immune–metabolic homeostasis. In addition, whether immune-metabolic pathways—such as the CD38–NAD^+^ axis—undergo functional reprogramming during rapid urate fluctuations and thereby contribute to inflammatory “rebound” phenomena remains a hypothesis requiring further investigation. Taken together, acute flares following rapid urate reduction are more plausibly explained by the combined effects of altered crystal dynamics and immune homeostatic remodeling rather than by a single mechanistic pathway.

To address these issues, future research should focus on three key areas: First, analyzing the dynamic characteristics of uric acid signaling across different populations, ages, and metabolic states to clarify its physiological and pathological thresholds through multi-omics and longitudinal cohort studies. Second, developing high-resolution *in vivo* imaging and crystal tracking technologies to enable real-time viSUAlization of AMSU formation and transformation, revealing its dynamic changes during asymptomatic periods. Third, establishing a multi-scale model of crystal-immune interactions to clarify the mechanisms linking crystal mobilization and immune rebound, thus providing a theoretical basis for safe and stable uric acid reduction strategies. Additionally, it is essential to promote mechanism-oriented clinical trial designs that integrate molecular markers and metabolic phenotypes with clinical endpoints, systematically evaluating the real benefits of dual-target interventions on multi-system diseases.
